# Immunohistochemical Expression of Ki-67, Dopamine D1 and Dopamine D2 Receptors in Meningiomas in a Tertiary Institution in Mexico

**DOI:** 10.7759/cureus.39826

**Published:** 2023-06-01

**Authors:** Luis A Rodríguez-Hernández, Jorge Navarro-Bonnet, Alma Ortiz-Plata, Juan P Gonzalez-Mosqueda, Pablo Martinez-Arellano, Metztli Calva-González, Marcos V Sangrador-Deitos, Michel G Mondragón-Soto, Diego Lopez Mena, Lesly Portocarrero-Ortiz

**Affiliations:** 1 Neurological Surgery, Instituto Nacional de Neurología y Neurocirugía Manuel Velasco Suárez, Mexico City, MEX; 2 Neurological Surgery, Instituto Nacional de Neurología y Neurocirugía Manuel Velasco Suarez, Mexico City, MEX; 3 Neuropathology, Instituto Nacional de Neurologia y Neurocirugía Manuel Velasco Suarez, Mexico City, MEX; 4 Neurology, Instituto Nacional de Neurología y Neurocirugía Manuel Velasco Suarez, Mexico City, MEX; 5 Psychiatry, Instituto Nacional de Neurología y Neurocirugía Manuel Velasco Suárez, Mexico City, MEX; 6 General Surgery, Centro Medico ABC, Mexico City, MEX; 7 Neuroendocrinology, Instituto Nacional de Neurología y Neurocirugía Manuel Velasco Suárez, Mexico City, MEX

**Keywords:** cancer, ki-67 expression, d2 receptors, dopamine receptors, atypical meningiomas

## Abstract

Objectives

Meningiomas (MNGs) are the most common intracranial tumors found in the adult population. While most intracranial MNGs may be surgically removed, a subset of patients remains ineligible for conventional treatment. This is either because of a lack of surgical access or due to atypical, anaplastic or invasive characteristics of the tumors. These patients may benefit from targeted therapies that focus on cell receptor expression. The aim of this study was to assess dopamine receptor (DR) and Ki-67 expression in the MGNs of patients treated with surgery in the Instituto Nacional de Neurología y Neurocirugía, Mexico.

Materials and methods

This study analyzed 23 patients with confirmed MNG diagnoses (10 female and 13 male (mean age: 44.5 years)) who had undergone surgical resection between 2010 and 2014 at our institution. In the collected samples, we performed analyses for Ki-67, Dopamine 1 and Dopamine 2 receptors’ expression.

Results

For the markers Ki-67, DR-D1 and DR-D2, the mean percentual expressions were 18.9%, 23.02% and 8.33%. No significant correlation was found between the expressions of these receptors and the studied MNG characteristics. The expression index of Ki-67 showed a significant relation with mean age (p = 0.03) and prolactin levels (p = 0.02).

Conclusions

Samples showed varied expressions of the studied receptors. Despite the difference in expressions between the markers, more studies are needed to confirm the findings. In contrast to previous studies, we could not find any relationship between D2-R and tumor characteristics.

## Introduction

Meningiomas (MNGs) are the most common primary CNS tumors found in adults [1]. Around 36.8% of all CNS tumor cases are diagnosed as MNGs, yet most are considered benign [2,3]. From a clinical standpoint, incidental diagnoses of MNGs are not uncommon and most cases are discovered at a later age (average 66 years) than other CNS neoplasms [4].

Most MNG patients are considered candidates for surgical resection or radiation therapy. The aim of these treatments is to both resect the tumor and eliminate the associated intracranial mass effect. Their success rates are high, and an elevated percentage achieves full resection [1].

Even when MNGs are fully resected, up to 60% may recur within 15 years [5] and require additional treatment. Moreover, up to 20% may show aggressive behavior [6]. While surgery and radiation therapy can be used again to treat recurrent cases, an important subset may become refractory. These cases, along with patients whose cases have demonstrated atypical, invasive or anaplastic behaviors, require systemic therapies.

Conventional systemic therapies and hydroxyurea have shown little to no effect on MNG treatment [7]. Considering this, several novel therapeutic agents, such as INF-alpha, VEFG and VEGF-R inhibitors, EGFR inhibitors, Imatinib and Somatostatin analogs, have been investigated for their use as systemic therapies in refractory cases.

Most chemotherapeutic agents have shown mild to moderate efficacy in disease stabilization and regression [7]. Therefore, there is a need for more research in target therapies that are based on the immunological and genetic information now available. Bevacizumab is an exception, as two Clinical Phase II trials have shown promising results on high-grade MNGs [8,9].

Considering the above. The need for more medical and immunological therapies for intracranial MNG treatment must not be understated. In fact, in the search for MNG management, there is still an open gate [10].

Dopamine receptors (DRs) are widely expressed in the central nervous system’s tissue. The five DR types can be broadly classified as D1-like family receptors (D1 and D5) and D2-like family receptors (D2, D3 and D4) [11]. D1-like family receptors upregulate the expression of adenylate cyclase making way for the proliferation of CAMP, while D2-like family receptors downregulate it via the MAPK and ERK pathways [11,12].

D1 and D2 receptors may play a role in cell regulation, as well as proliferation in tumor cells of central nervous system neoplasms. In vitro D1 and D2 receptor activation using dopamine and select agonists, such as Bromocriptine, have successfully reduced the proliferation rate of pituitary tumor cells [13,14] and MNG tumor cells [15].

Ki-67 is an important marker known for assessing tumor progression in several types of cancer [16,17]. In the case of MNGs, it has been associated with worse prognoses and overall survival [18]. Several studies point toward Ki-67’s potential use as a biomarker for disease progression, recurrence and time of recurrence [19,20].

This study’s aim was to assess expressions of Dopamine 1 and 2 receptors and Ki-67 in MNGs and compare them against social and biochemical risk factors.

## Materials and methods

Inclusion

This was a retrospective, observational, transversal, analytic study. It analyzed tumor samples obtained from 23 randomly selected adult patients with clinical and anatomopathological diagnoses of MNG. These patients had undergone surgical resection at the Neurosurgery Department of the National Institute of Neurology and Neurosurgery “Manuel Velasco Suarez” in Mexico City between January 2010 and December 2015. Patients’ medical records were obtained for demographic and clinical variables and Serum Prolactin, LH and FSH levels were attained prior to surgical resection.

Tissue samples were properly obtained, processed and classified according to the WHO classification’s criteria for histological subtype and tumor grade. Patients with tissue samples postoperatively classified as inconclusive or diagnosed as other CNS tumors were excluded.

Immunohistochemistry

MNG tissue samples were embedded in paraffin blocks, sectioned into slices 4µm thick, placed in lamellae with Poly-L-lysine and deparaffinized and rehydrated in alcohol solutions of descending concentration gradients (absolute, 96%, 80% and 70%).

Tissue samples were later prepared for antigenic recovery and stained for the following primary antibodies: anti-Ki-67 clone MIB-1 (Dilution 1: 100, Dako, Denmark, catalog number M7240) for one hour and anti-dopamine D1 receptor (Dilution 1: 500, Abcam, Cambridge, England, catalog number ab-20066) overnight at 4°C.

For two minutes, counterstaining was performed with hematoxylin rinsed in tap water, and the stain was rotated in a 0.5% lithium carbonate solution for 10 seconds. Finally, the lamellae were dehydrated in 70%, 80% and 96% alcohol solutions and absolute OH-xylol and xylol before being mounted in a resin within coverslips (Entellan, Merck, Darmstadt, Germany).

Biopsies were first observed with light microscopy (Olympus BH2). The analyses of expressions of the different markers (Ki-67, RD-D1 and RD-D2) were then performed using a Nikon Eclipse E200 microscope (Tokyo, Japan) at a 40x magnification, which acquired images with the Lagine program Q-Capture-Pro 7 (2010) at a rate of 10 fields per sample. Quantitative analyses of the expressions were performed through the ImageJ® 1.49V program (Wayne Raasband, National Institute of Health, USA, http//imageJ.nih.gov/IJ).

Statistical analysis

Findings on the Ki-67, RD-D1 and RD-D2 expressions were statistically evaluated using analysis of variance (ANOVA) and a T-test with a CI of 95% and p established at <0.05 to look for associations and correlations of these markers, serum biomarkers (PRL, LH and FSH) and the MNG’s location, along with histological subtype.

Ethics

No personal identifying information was obtained. All patients were required to sign an informed consent form before their inclusion into the study. The study was designed based on the specifications and ethical principles for medical research involving human subjects and was submitted for review and approved by the internal Institutional Research Committee (protocol number 20/16).

## Results

Of the 23 patients included in the study, 13 were male (56.52%), which corresponded to a male-female ratio of 1.3:1. The mean patient age was 44.52 years (IQR: 26-71 years). The average BMI was 28.42 kg/m^2^ (IRQ: 19.5-39.8 kg/m^2^). All patients underwent successful surgical resection, and 20 out of 23 (86.95%) were either diagnosed with a residual tumor or experienced recurrence within five years (see Table 1 for further information).

**Table 1:** Clinical and demographic characteristics of patients included in the study

Categories	
Age	44.52 years (IQR: 26-71)
Male Sex	13 (56.52%)
BMI	28.42 kg/m^2^ (IRQ: 19.5-39.8 kg/m^2^)
Residual or Recurrent Tumor	20 (86.95%)
Meningioma Location	
Posterior Fossa	9 (39.13%)
Anterior Skull Base	7 (30.43%)
Mid-Skull Base	5 (21.73%)
Convexity	2 (8.69%)
WHO Classification	
Typical	16 (69.56%)
Anaplastic	4 (17.39%)
Atypical	3 (13.04%)
Cell Markers Expression	
Ki-67 Positive Expression	21 (91.3%)
Average Percentage of Ki-67 Cell Expression	18.9% (7-50.8%)
D1-R Positive Expression	23 (100%)
Average Percentage of D1-R Cell Expression	23.02% (2.9%-65.0%)
D2-R Positive Expression	20 (86.95%)
Average Percentage of D2-R Cell Expression	8.33% (0.1%-45.8%)

Considering the location, MNGs were mostly found in the posterior fossa (9/23; 39.13%), followed by the anterior skull base (7/23; 30.43%), the mid-skull base (5/23; 21.73%) and the convexity (2/23; 8.69%). According to WHO Classification (69.56%; 16/23) were classified as typical, (17.39%; 4/23) were anaplastic and (13.04%; 3/23) were atypical.

Regarding biochemical measurements, 14 patients (seven male and seven female) were found to have elevated levels of Serum Prolactin (mean: 27.67 mIU/ml), LH (mean: 6.29 mIU/ml) and FSH (mean: 11.2 mIU/ml).

All samples (100%) were positive for D1-R expression, 20/23 samples (86.95%) were positive for D2-R expression and 21/23 samples (91.3%) were positive for Ki-67 expression. The Ki-67 mean percentual expression index was 18.9% (range: 7.0-50.8), which was higher in men than women (21.63% vs. 15.34%). Moreover, the average RD-D1 mean percentual expression index was 23.02% (range: 2.9%-65.0%): higher in men (27.01%) than in women (17.83%). Furthermore, the average RD-D2 expression index was 8.33% (range: 0.1%-45.8%), which was also higher in men (9.35%) than in women (7.0%).

Statistical analysis was performed to assess whether the expressions of Ki-67, RD-D1 and RD-D2 had significant relationships with any of the variables or between the same markers. Statistically significant associations were discovered between age and Ki-67 expression (p = 0.03) and Serum Prolactin levels and Ki-67 expression (p = 0.02) (see Table 2).

**Table 2:** Statistical significance of associations between cell expression of Ki-67, D1-R and D2-R and clinical characteristics. FSH: follicle-stimulating hormone; LH: luteinizing hormone; PRL: prolactin; BMI: body mass index

Category	Ki-67	D1-R	D2-R
Sex	P=0.828	P=0.321	P=0.494
Location of the Meningioma	P=0.658	P=0.762	P=0.873
FSH Levels	P=0.572	P=0.713	P=0.825
LH Levels	P=0.421	P=0.799	P=701
PRL Levels	P=0.024	P=0.145	P=0.833
BMI	P=0.290	P=0.110	P=0.638

The following conveyed no statistically relevant differences: sex (Ki-67 (p = 0.828)), (D1-R (p = 0.321)), (D2-R (p = 0.494)); the location of the MNG (Ki-67 (p = 0.362)), (D1-R (p = 0.268)), (D2-R (p = 0.670)); the histological grade according to the WHO classification of 2016 (Ki-67 (p = 0.658)), (D1-R (p = 0.762)), (D2-R (p = 0.873)); FSH levels (Ki-67 (p = 0.572)), (D1-R (p = 0.713)), (D2-R (p = 0.825)); LH levels (Ki-67 (p = 0.421)), (D1-R (p = 0.799)), (D2-R (p = 0.701)); PRL levels (Ki-67 (p = 0.024)), (D1-R (p = 0.145)), (D2-R (p = 0.833)) and BMI (Ki-67 (p = 0.290)), (D1-R (p = 0.110)), (D2-R (p =0.638)).

There was no significant relationship between the studied cell markers: Ki-67 did not have a significant relationship with D1-R (p = 0.955) or D2-R (p = 0.416). Moreover, no such relationship existed between D1-R and D2-R (p = 0.539).

## Discussion

Surgery, radiation therapy and chemotherapy remain the cornerstone for treating patients with MNGs [21]. While most cases may be managed or cured by these methods, a small number of cases - those that resist treatment or are recurrent - remain a therapeutic challenge.

This study, while reduced in scope, does provide interesting facts regarding the expressions of DRs and Ki-67 in MNGs. The study results, which were obtained from a small subset of Mexican patients with intracranial MNGs, did not differ from the data of previous studies. Furthermore, our population findings concerning age and histological type did not contrast with other published MNG studies.

Previously, Ki-67 and D2-R have been proposed as markers to further classify and understand MNGs to perform target therapies that enhance patient outcomes in recurrent or otherwise complicated cases.

As mentioned before, Ki-67 is a cellular marker of cellular proliferation. Its expression increases during the second half of the S phase; it reaches its peak during the G2 and M phases and then slowly decreases until it becomes untraceable in the G0 and G1 phases [17]. Ki-67 has previously been analyzed, specifically in MNGs. Moreover, it has been demonstrated to correlate positively with severer prognoses and a higher WHO histological grade [18].

Several associations between Ki-67 expression and MNGs remain under study. However, evidence continues to be mixed on Ki-67 expression in recurrent tumors [18]. While several studies [20,22,23] show a positive relationship between the Ki-67 index and recurrence, others have not [24]. Also, research shows that due to its role in cellular proliferation, Ki-67 expression may be enhanced in a tumor’s peripheral area as opposed to its core [25].

A study by Pavelin et al. [26] analyzed the variables of Ki-67 and P53 as tumor markers in MNGs. The study found a significant correlation between Ki-67 and the MNG subtype as well as size. By comparison, our study did not set a cutoff value for Ki-67 proliferation, and its index was used as a continuous variable instead. Also, unlike the study by Pavelin et al. [26], ours found a statistically significant relationship between Ki-67 expression and age.

We also found a statistically significant relationship between Serum Prolactin levels and Ki-67 expression. We hypothesize that this association can be attributed to an underlying relationship between the level of activity in the downregulation of dopaminergic pathways that closely mimic the level of overall activity in the tumor.

DRs have been found to modulate proliferation activity and mitosis in MNG cells via intracellular and extracellular pathways. In vitro studies have shown that D1 receptors activated by micromolar concentrations of bromocriptine (used in those concentrations as a D1 agonist), dopamine and other D1 agonists can lead to reduced proliferation activity of MNG cells [15].

On the other hand, dopamine and dopamine 2 agonists, like haloperidol, have been shown to modulate cell death via dopamine 2 receptor pathways [13,14,27]. Moreover, a case report on a patient with a recurrent sellar MNG with symptoms of hyperprolactinemia showed that Cabergoline treatment (a D2 agonist) slowed the tumor’s progression for 11 years [28]. This is additional evidence supporting further study on the dopaminergic pathway in MNGs.

The presence of DRs in MNG samples has been successfully studied using immunohistochemistry, polymerase chain reaction and Northern-Blot [12,29]. Since immunohistochemistry is one of the cheapest and more readily available methods, we decided to use this technique - albeit performing a quantitative analysis rather than a qualitative one.

A study by Trott et al. found a relationship between the positive expression of D2-R and MNGs [12]. Like our study, it discovered no statistically significant relationships between D2-R expression and patient and tumor characteristics. This is the closest study to ours concerning patient selection and methods; however, it used a qualitative analysis [12].

The fact that D2 receptors were found in more than 90% of the analyzed MNGs in both studies, including histologically malignant ones, points toward this receptor’s potential use as a potential tool for the treatment of these tumors.

Several limitations were found while conducting this study. Due to its small sample size, there were numerous key differences between our study population and those of previous studies. Most notably, we believe the gender disproportion (skewed toward males) may be explained by this fact. We also believe some population characteristics were affected by the study’s location. Since the research took place in a tertiary institution, many of the cases were considered either surgically or medically challenging due to considerations other than the WHO Classification. Some, such as the surgical dexterity required to complete surgery or the anatomical location in the skull base, may explain our remarkably higher incidence rate (86.9%) of residual or recurrent MNGs.

## Conclusions

The study evaluated the presence of Ki-67 and D1 and D2 receptors in intracranial MNG samples. A considerable minority showed Ki-67 and D2 receptor expression. However, no statistically significant relationship could be found between the markers and age, location or histological grade.

While existent information supports continuing research on the potential use of Ki-67 as a prognostic marker in MNGs and DRs as indicators for potential treatment, more research is needed to eventually consider using these markers as routine.

Further studies, using a larger population, may help to better characterize the associations of DRs and Ki-67 in MNGs in patients with recurrent MNG or MNGs that cannot otherwise be surgically removed or treated with conventional chemotherapy.
